# Three decades of the Mediterranean diet pyramid: A narrative review of its history, evolution, and advances^☆^

**DOI:** 10.1016/j.ajcnut.2025.04.036

**Published:** 2025-05-13

**Authors:** Frank B Hu, Greg Drescher, Antonia Trichopoulou, Walter C Willett, Miguel A Martínez-González

**Affiliations:** 1 Department of Nutrition, Harvard T.H. Chan School of Public Health, Boston, MA, United States; 2 Department of Epidemiology, Harvard T.H. Chan School of Public Health, Boston, MA, United States; 3 Channing Division of Network Medicine, Department of Medicine, Harvard Medical School, Brigham and Women’s Hospital, Boston, MA, United States; 4 The Culinary Institute of America, Hyde Park, NY, United States; 5 Center for Public Health Research and Education, Academy of Athens, Athens, Greece; 6 Medical School, University of Athens, Athens, Greece; 7 Centro de Investigación Biomédica en Red Fisiopatología de la Obesidad y la Nutrición (CIBEROBN), Institute of Health Carlos III, Madrid, Spain; 8 Department of Preventive Medicine and Public Health, Instituto de Investigación Sanitaria de Navarra, University of Navarra, Pamplona, Spain

**Keywords:** Mediterranean diet, olive oil, sustainability, cohort studies, metabolomics, cardiovascular disease, nutritional epidemiology, randomized controlled trials

## Abstract

**Background:**

The Mediterranean diet (MedDiet) pyramid was officially published in the *American Journal of Clinical Nutrition* in 1995. Since then, our understanding of the role of the MedDiet and its role in reducing risk of chronic diseases has grown substantially.

**Objectives:**

This article aims to provide a narrative review of the historical context of the MedDiet and its environmental impact, summarize health-related evidence from the past 3 decades, and explore its practical applications and cultural adaptations.

**Methods:**

Relevant studies were identified through searches of PubMed and other major databases. We prioritzed publications from the past 3 decades, while also including influential earlier studies where appropriate. Evidence was synthesized qualitatively to summarize key findings and identify gaps for future research.

**Results:**

A large body of evidence from prospective cohort studies, randomized controlled trials, and mechanistic studies consistently supports the benefits of the MedDiet for the prevention of chronic diseases, particularly cardiometabolic diseases and the improvement of healthy aging. Growing evidence demonstrates that the MedDiet promotes favorable changes in circulating metabolites and gut microbiome composition, providing novel insights into biological mechanisms underlying its health benefits and informing the development of precision nutrition strategies. The MedDiet aligns with the principles of the planetary health diet recommended by the EAT-Lancet Commission, which aims to promote both human health and environmental sustainability. The development of the MedDiet pyramid 30 y ago inspired the creation of the Asian, African, and Latin-American heritage diet pyramids.

**Conclusions:**

Over the past 3 decades, substantial evidence has accumulated to strongly support the benefits of the MedDiet in preventing chronic diseases and promoting healthy aging. Despite robust evidence, further studies are needed to evaluate the long-term effectiveness and adaptability of the MedDiet across diverse populations, cultural settings, and food environments.

## Introduction

The Mediterranean diet (MedDiet) has long been recognized for its health benefits and cultural significance [[1], [2], [3]]. A special supplement of the *American Journal of Clinical Nutrition*, published in June 1995, introduced the MedDiet pyramid graphic (Figure 1 [4]), along with 17 reviews that generated global interest in the traditional MedDiet. This milestone publication has profoundly shaped nutrition research, public communications on healthy eating, and dietary guidelines worldwide over the past 3 decades. The MedDiet was designated by the United Nations Educational, Scientific, and Cultural Organization in 2010 as part of the intangible cultural heritage of humanity [1,2].

**FIGURE 1:**
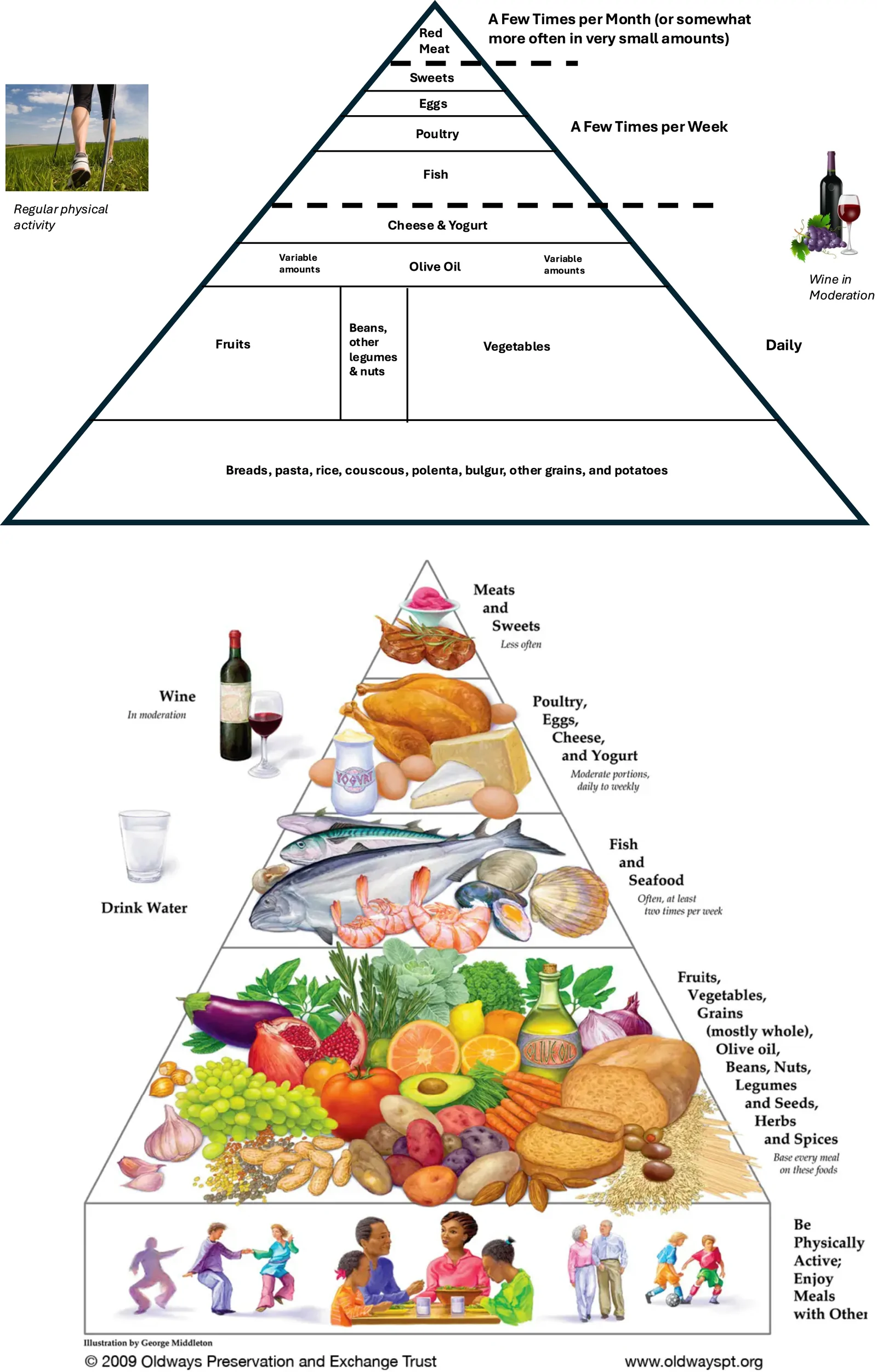
The original Mediterranean diet pyramid published in 1995 in the *American Journal of Clinical Nutrition* (upper panel) [4] and an updated version (2008) developed by Oldways Preservation and Exchange Trust in collaboration with the WHO and the Harvard T.H. Chan School of Public Health (lower panel). At the base of the pyramid are the core foods eaten at almost every meal, namely plant foods including vegetables, fruits, nuts, whole grains, olive oil, and spices. Water is the main beverage for everyone. For individuals who choose to consume alcohol, it is consumed in moderation and typically with a meal. Moving upward is seafood, which is eaten at least twice a week. Moving further up are foods eaten on a daily to weekly basis, including eggs, poultry, and dairy products (yogurt and traditional cheese). At the top of the pyramid are foods consumed less frequently, such as red meat, processed meat products, and sweets, which are eaten no more than a few times a month. WHO, World Health Organization.

On 21 November 2024, the Spanish National Research Council, in collaboration with the International Olive Council and the International Center for Advanced Agronomic Studies, hosted a symposium in Madrid, Spain, to commemorate the 30th anniversary of the official publication of the MedDiet pyramid. The event reviewed key advances, challenges, and future directions in MedDiet research and cultural adaptations to diverse populations. Building on the discussions and insights from this symposium, this narrative review aims to summarize the historical context of the MedDiet, the evolution of evidence in its role on human and planetary health, and the translation of this healthy eating pattern to practical actions through cross-disciplinary and cross-cultural collaborations.

## Literature Search and Synthesis

Relevant studies were identified through searches of PubMed and other major databases (Embase and Cochrane Library). We focused on publications from the past 3 decades, with key earlier studies included where appropriate. Studies were selected based on their relevance to the role of the MedDiet for chronic disease prevention, environmental sustainability, and practical applications. Evidence was synthesized qualitatively to summarize key findings and identify gaps for future research.

## Historical Context

The traditional MedDiet pattern has long been characteristic of the regions surrounding the Mediterranean Sea, including parts of Southern Europe, North Africa, and the Eastern Mediterranean. Since antiquity, it has been defined by a moderate-to-high fat content and heavy reliance on plant-sourced ingredients, including fruits and vegetables, whole grains, legumes (pulses), nuts and seeds, and an abundance of olive oil [4]. However, the health aspects of this dietary pattern only began to gain recognition in the scientific and public health communities in Europe and the United States after the Second World War. In 1948, the Rockefeller Foundation commissioned a study led by Leland Allbaugh, which documented the healthfulness of the dietary pattern of post-war Crete [5]. The Seven Countries Study initiated by Ancel Keys in 1956 was the first large-scale epidemiological study to systematically examine the relationship between diet and cardiovascular disease (CVD) risk across multiple populations [6,7]. This ecological study demonstrated remarkably low rates of coronary artery disease (CAD) in Mediterranean populations, especially on the island of Crete, Greece, as well as in Japan, compared to Northern European countries and the United States.

Despite early awareness of the healthy “Mediterranean Diet,” this was vastly overshadowed in the 1980s by a widespread belief that reducing total fat—to 30% of energy [8] or 20% or lower [9,10]—should be a key pillar of dietary guidance for reducing the incidence of chronic disease. Thereafter, building on the earlier work of Ancel Keys, AntoniaTrichopoulou rekindled interest in the traditional MedDiet with her research on adherence to the MedDiet and reduced risk of mortality in Greek populations [11,12]. These prospective cohort studies operationalized the MedDiet pattern by developing a scoring system that captures the key aspects of this dietary pattern, enabling large-scale epidemiologic studies into its long-term health benefits.

It was not until the early 1990s that a collaboration of individuals and organizations emerged—across academic disciplines and professional groups— and began to challenge the widespread dietary recommendation promoting low-fat diets for better health, laying the foundations for the subsequent increase in scientific, public policy, media, and consumer interest in the MedDiet. This collaboration, co-led by scientists from the Harvard T.H. Chan School of Public Health, the Nutrition Unit of the WHO Regional Office for Europe, the Athens School of Public Health, the National Institute of Nutrition in Rome, and the Boston-based nonprofit Oldways, resulted in 2 international academic conferences held in the Boston area in 1993 and 1994. These efforts culminated in the release of the MedDiet pyramid and a 1995 special supplement of the *American Journal of Clinical Nutrition*, which was dedicated to cultural, culinary, and health aspects of the MedDiet.

One of the key findings from this collaboration, based on a review of the scientific literature and other data on nutrition and health in the Mediterranean regions, was that various health outcomes, including chronic disease rates and life expectancy in these regions, were strongly influenced by close proximity to extensive olive cultivation. This initiative, which led to the development of the MedDiet pyramid, established an evidence-based definition of “the Mediterranean Diet” from the outset. It was grounded on the available evidence reflecting the food patterns of Crete, much of the rest of Greece, and southern Italy in the early 1960s as a model for health-promoting dietary practices [[12], [13], [14]].

The 1995 *American Journal of Clinical Nutrition* article on the MedDiet model emphasized that although the MedDiet is often associated with specific regions, variations exist across different parts of the Mediterranean, including Italy, France, Lebanon, Morocco, Portugal, Spain, Syria, Tunisia, Turkey, and beyond [4]. It highlighted the need for greater precision in defining the MedDiet model. Notably, differences in the food, diet, and health indicators between southern and northern Italy in the early 1960s illustrated the importance of linking the MedDiet to food patterns rather than political or geographical boundaries. In southern Italy, olive oil was the primary fat source, and meat consumption was low, whereas in northern Italy, dairy fats were more prevalent, and meat was higher. In Turkey, olive cultivation is concentrated in the coastal areas, whereas consumption of animal fats is much higher in the interior north and east. In France, olive oil is widely consumed in Provence, in contrast to the regular use of animal fats in the north. These variations underscore the diversity of dietary patterns even in Mediterranean populations.

The MedDiet pyramid stood in sharp contrast to the influential USDA food guide pyramid, which emphasized reducing all types of fat whereas promoting high consumption of carbohydrates along with dairy [15]. In contrast, the MedDiet pyramid highlighted the quality of fats, emphasizing olive oil as the primary fat source and prioritizing plant-based proteins such as nuts and legumes over animal-based proteins, particularly red meat. Following its launch, the MedDiet pyramid became a globally recognized symbol, which has shaped nutrition research agendas, dietary guidelines, and public health recommendations as well as food industry practices, restaurant menus, and home cooking behaviors worldwide.

The MedDiet collaboration of the early 1990s demonstrated the value of partnerships among scientists, public health leaders, and culinary professionals in advancing nutrition research and practice. It has also spurred greater interest in adapting the principles of the MedDiet to other traditional dietary patterns, such as those of Asia, Africa, and Latin America. These efforts have led to the development of Asian, African, and LatinAmerican Heritage Diet Pyramids [16]. Moreover, the expansion of olive cultivation and olive oil production in regions such as the United States, Latin America, Asia, Eastern Europe, and the Middle East reflects a growing global interest in this staple of Mediterranean cuisine.

Cultural models for healthy eating, including MedDiet, need to be understood in their historical contexts and adapted to new scientific evidence on diet and health. Changes in food systems and eating habits, increasing sedentary lifestyles, and rising obesity rates, especially among children in many Mediterranean countries and globally [17,18], underscore the need to revisit and refine traditional dietary patterns. These global trends underscore the importance of updating culturally rooted dietary patterns like the MedDiet to better address contemporary public health challenges.

### Evidence from large cohort studies

Advances in the field of nutritional epidemiology have been propelled by numerous large prospective cohort studies conducted worldwide. These cohorts offer significant advantages, including large sample sizes, extended follow-up durations, and comprehensive assessments of diet and lifestyle factors. Systematic reviews and meta-analyses of findings from these cohorts provide opportunities to integrate data from diverse populations, resulting in even larger sample sizes and enhanced statistical power [19].

The cardiovascular health benefits of the MedDiet were first identified in the Seven Countries Study led by Ancel Keys who was the first to coin the term “Mediterranean Diet,” The study reported remarkably low rates of CAD mortality on the Greek island of Crete compared to those in Northern Europe and the United States [6,7,20]. However, as an ecological study, these findings may be susceptible to confounding by other factors, such as lifestyles and environmental influences. Since then, the associations between adherence to the MedDiet and a wide range of health outcomes have been investigated in numerous large prospective cohorts. Among 22,043 Greek adults, adherence to the MedDiet was assessed using a 10-point MedDiet score. Participants were subsequently followed up for a median of 44 mo [21]. Higher adherence (a score of 6–9 compared with 0–3) was associated with lower total mortality [hazard ratio (HR) per 2-point increment: 0.75; 95% confidence interval (CI): 0.64, 0.87), CVD mortality (HR: 0.67; 95% CI: 0.47, 0.94), and cancer mortality (HR: 0.76; 95% CI: 0.59, 0.98).

The Nurses’ Health Study (NHS) and the Health Professionals Follow-Up Study (HPFS) are among the largest and longest-analyzing investigations of diet and health globally [22]. Beyond their large sample sizes and follow-up periods exceeding 3 decades, they maintain exceptionally high long-term follow-up rates (>90%). Moreover, detailed data on diet and lifestyle factors have been systematically collected every 2–4 y, allowing for a high level of control for potential confounding. The repeated dietary assessments are particularly valuable for minimizing measurement errors associated with self-reported dietary intake, capturing long-term dietary habits more effectively, as well as opportunities to examine dynamic changes in diet in relation to subsequent disease risk [23].

In the NHS involving 74,886 females followed for 20 y, higher adherence (highest compared with lowest quintiles) to the alternative MedDiet (aMED) score was associated with a 29% lower CAD risk [relative risk (RR): 0.71; 95% CI: 0.62, 0.82] and a 13% lower stroke risk (RR: 0.87; 95% CI: 0.73, 1.02) [24]. Also, a comparative analysis conducted in the NHS and HPFS showed that adherence to healthy eating index-2015, aMED, healthful plant-based diet index, and alternate healthy eating index (AHEI) was similarly associated with lower risk of CVD [25] and mortality [26] (Figure 2 [25,26]). These findings highlight the convergence of diverse healthy dietary patterns in reducing risk of chronic diseases and mortality. Despite some differences, these dietary patterns share many common components that contribute to their health benefits.

**FIGURE 2:**
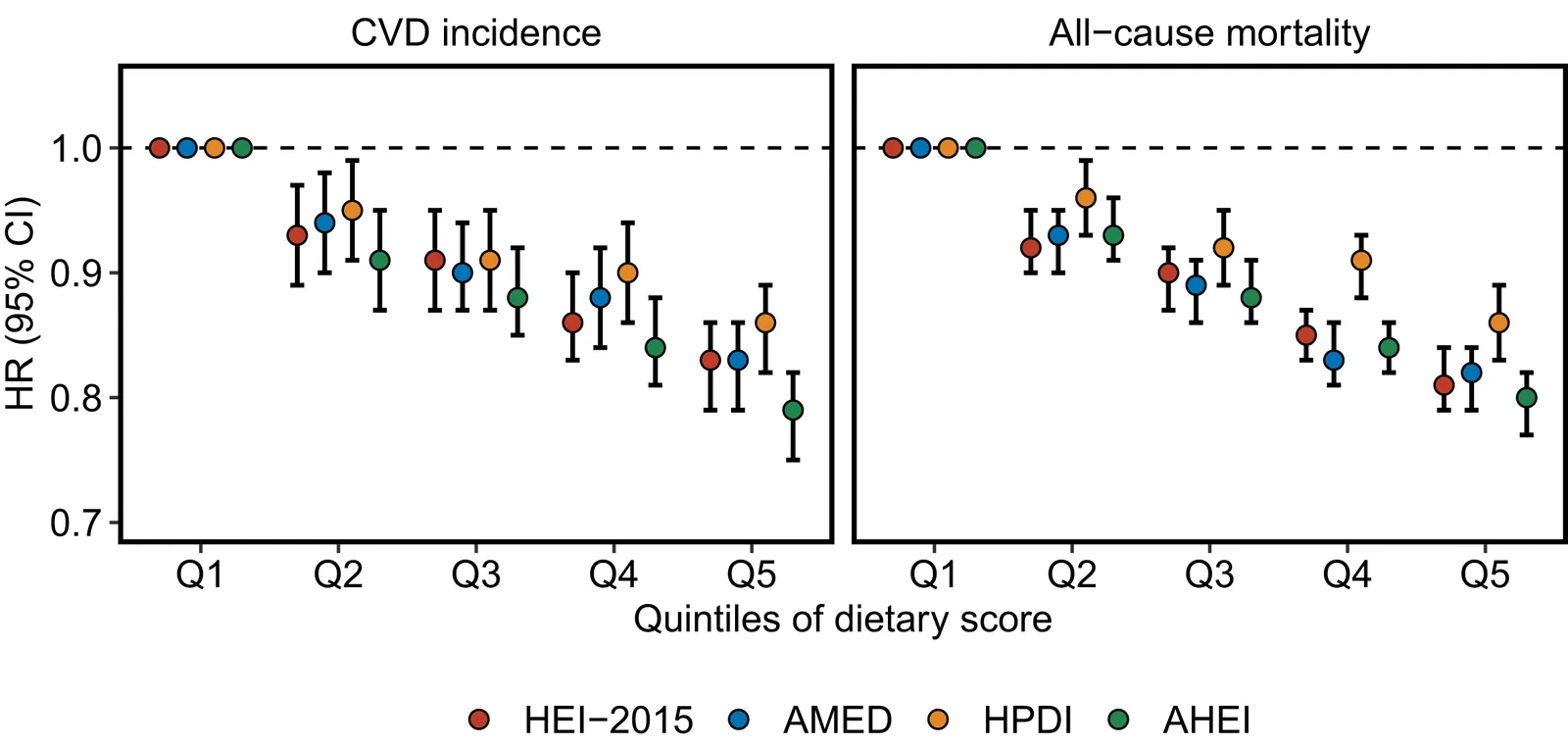
Hazard ratios of cardiovascular disease and all-cause mortality according to quintiles of the HEI-2015, aMED score, HPDI, and AHEI. The nurses’ health study and health professionals’ follow-up study included 209,133 participants [25,26]. Results shown are hazard ratios (HR) and 95% confidence intervals (CIs) estimated from multivariable-adjusted Cox regression models stratified by age and calendar year, and adjusted for BMI, race and ethnicity, marriage status, living status, family history of myocardial infarction, menopausal status, multivitamin use, aspirin use, total energy intake, smoking status, alcohol drinking, physical activity, history of hypertension, and history of hypercholesterolemia. Results for all-cause mortality were additionally adjusted for family history of diabetes and family history of cancer. For CVD incidence analysis, 23,366 incident cases among 209,133 participants were included. For all-cause mortality analysis, 54,163 deaths among 119,315 participants were included. AHEI, alternate healthy eating index; aMED, alternative Mediterranean diet; BMI, body mass index; CVD, cardiovascular disease; HEI-2015, healthy eating index, 2015; HPDI, healthful plant-based diet index; Q1–Q5, quintiles.

Analyzing repeated dietary measures in the NHS and HPFS, increasing adherence from baseline to the first 4-y follow-up was associated with lower CVD risk during the next 20 y 7% (95% CI: 1, 12%) for the AHEI, and 9% (95% CI: 3, 14%) for the aMED score [27]. A decrease in diet quality scores was associated with a significantly elevated risk of CVD in subsequent time periods. In the NHS and HPFS, improved diet quality over 12 y, assessed by the AHEI, aMED, and dietary approaches to stop hypertension scores, was associated with an 8–17% lower risk of mortality for every 20-percentile increase in diet quality scores [28]. In contrast, worsening diet quality was associated with a 6–12% higher risk. Participants who maintained a high-quality diet had a 9–14% lower risk of death compared to those with consistently low scores.

Adherence to MedDiet has also been associated with a lower risk of type 2 diabetes (T2D). In the Women's Health Study, a higher aMED score was associated with a 30% RR reduction in T2D over a 20-y period, largely mediated by biomarkers of insulin resistance, BMI (in kg/m^2^), lipoprotein metabolism, and inflammation [29]. In a meta-analysis of 14 prospective cohort studies, including 410,303 participants and 41,466 cases of T2D, greater adherence was associated with a 21% lower risk of T2D (RR: 0.79; 95% CI: 0.72, 0.88), and each 2-point increase in the adherence score was associated with a 14% reduction in risk (RR: 0.86; 95% CI: 0.82, 0.91) [30]. These associations remained robust across subgroup analyses, including geographical regions, in a linear dose-response pattern.

Adherence to the MedDiet has been consistently associated with a lower risk of total and individual cancers. In another meta-analysis of 117 case-control and cohort studies, including over 3.2 million participants, higher adherence to the MedDiet was significantly associated with a 13% lower risk of cancer mortality (RR: 0.87; 95% CI: 0.82, 0.92), 25% lower risk of all-cause mortality among cancer survivors (RR: 0.75; 95% CI: 0.66, 0.86), and lower risks for several site-specific cancers, including colorectal (RR: 0.83; 95% CI: 0.76, 0.90), breast (RR: 0.94; 95% CI: 0.90, 0.97), head and neck (RR: 0.56; 95% CI: 0.44, 0.72), gastric (RR: 0.70; 95% CI: 0.61, 0.80), liver (RR: 0.64; 95% CI: 0.54, 0.75), bladder (RR: 0.87; 95% CI: 0.76, 0.98), and respiratory cancers (RR: 0.84; 95% CI: 0.76, 0.94) [31]. No significant associations were observed for hematological, esophageal, pancreatic, or prostate cancers.

Adherence to the MedDiet has been associated with improved cognitive health and lower risk of neurodegenerative diseases. In a meta-analysis including 31 cohort studies and 5 randomized controlled trials (RCTs), high adherence to the MedDiet was associated with a 25% lower risk of mild cognitive impairment (RR: 0.75; 95% CI: 0.66, 0.86) and a 29% lower risk of Alzheimer’s disease (RR: 0.71; 95% CI: 0.56, 0.89). In RCTs, higher adherence to the MedDiet was linked to improved episodic memory and working memory [32]. Although the evidence suggests a protective role of the MedDiet against cognitive decline, further long-term RCTs are needed to strengthen these findings.

Olive oil consumption is considered a hallmark of the traditional MedDiet. In a meta-analysis of 27 studies, including 24 prospective cohort studies and 3 reports from 1 RCT, every additional 25 g/d of olive oil consumption was associated with a 16% reduced risk of CVD (RR: 0.84; 95% CI: 0.76, 0.94), a 22% lower risk of T2D (RR: 0.78; 95% CI: 0.69, 0.87), and an 11% reduction in all-cause mortality (RR: 0.89; 95% CI: 0.85, 0.93). However, no significant association was observed between olive oil consumption and cancer risk (RR: 0.94; 95% CI: 0.86, 1.03) [33].

Although olive oil intake is much lower in the United States population compared to the Mediterranean populations, the data from the NHS and HPFS support a significant association between higher olive oil consumption and lower risk of chronic disease and mortality. In particular, higher olive oil intake (>0.5 tablespoons/d or >7 g/d) was associated with a 14% lower risk of CVD (HR: 0.86; 95% CI: 0.79, 0.94) and an 18% lower risk of CAD (HR: 0.82; 95% CI: 0.73, 0.91), with stronger inverse associations observed when margarine, butter, or mayonnaise were replaced with olive oil [34]. In addition, consuming >7 g/d of olive oil was associated with a 10% lower risk of T2D (HR: 0.90; 95% CI: 0.82, 0.99), and substitutions of olive oil for stick margarine, butter, or mayonnaise were associated with a 5–15% lower risk of T2D [35]. In terms of cause-specific mortality, higher olive oil intake was associated with 19% lower CVD mortality (HR: 0.81; 95% CI: 0.75, 0.87), 17% lower cancer mortality (HR: 0.83; 95% CI: 0.78, 0.89), 29% lower neurodegenerative disease mortality (HR: 0.71; 95% CI: 0.64, 0.78), and 18% lower respiratory disease mortality (HR: 0.82; 95% CI: 0.72, 0.93) [36]. Moreover, consuming ≥7 g/d of olive oil was associated with a 28% lower risk of dementia-related death (HR: 0.72; 95% CI: 0.64, 0.81), independent of diet quality or genetic predisposition [37].

In a recent analysis of the NHS and HPFS cohorts, higher intake of total plant-based oils, both with and without olive oil, was associated with a lower risk of mortality, whereas higher butter consumption was linked to increased mortality [38]. Among individual oils, higher intakes of canola, soybean, and olive oils were associated with lower mortality, with olive oil providing the greatest benefit. These findings suggest that replacing butter with plant-based oils may significantly reduce risk of premature death. Although these findings consistently support the potential benefits of olive oil in reducing cardiometabolic, neurodegenerative, and overall mortality outcomes, these studies did not differentiate between extra-virgin olive oil (EVOO) and refined olive oil. Also, due to the observational nature of the study design, residual confounding by a healthier diet and lifestyles associated with higher olive oil intake cannot be completely ruled out.

### Evidence from large, randomized intervention trials

Although large prospective cohort studies have yielded substantial knowledge on the relationship between adherence to the MedDiet and health outcomes, large RCTs with a long follow-up period and hard clinical endpoints are needed to provide the strongest causal inference. However, such trials are often impractical due to financial and logistical constraints. Over the past 3 decades, only a few large RCTs have examined the effects of the MedDiet in primary or secondary prevention of CVD, and they represent some of the most significant advances in understanding the role of MedDiet in chronic disease prevention.

The “PREvención con DIeta MEDiterránea” (PREDIMED) trial conducted in Spain has provided the best available scientific evidence on the benefits of the MedDiet for the prevention of CVD. PREDIMED was a primary prevention trial which randomly allocated high-risk participants (aged 55–80 y, 57% females) to 3 groups, a MedDiet supplemented with EVOO (*n* = 2543); a MedDiet supplemented with tree nuts (*n* = 2454); or a control group randomly assigned to a low-fat diet (*n* = 2450). The primary endpoint was a composite of nonfatal stroke, nonfatal myocardial infarction, or any cardiovascular death. After 5 y of intervention, compared to the control group, there was a 31% relative reduction in the risk of the primary endpoint in the MedDiet group supplemented with EVOO, and a 28% RR reduction with the MedDiet group supplemented with mixed nuts [39] (Figure 3). Additional benefits of the MedDiet intervention, as compared to the low-fat diet control group, were observed for other endpoints, including T2D, peripheral artery disease, atrial fibrillation, and breast cancer [40]. It should be noted that although the low-fat control group was advised to follow a diet with <30% of energy from fat, their actual fat intake remained around 37% of total energy. Therefore, despite receiving repeated education and counseling to follow a low-fat diet, the actual profile of the control group could be characterized as a usual care group rather than a true low-fat group. The primary distinction between the control and intervention groups was the supplementation of EVOO or nuts in the MedDiet groups.

**FIGURE 3:**
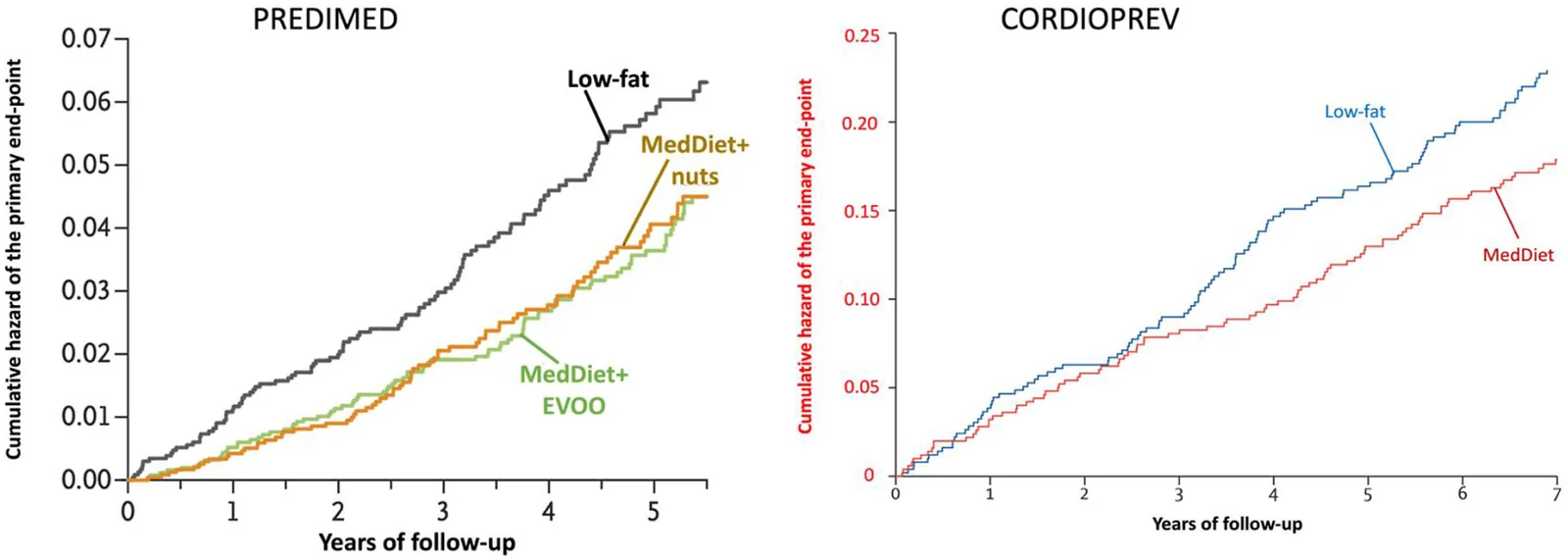
Cumulative hazard of the cardiovascular primary endpoint in the PREDIMED trial (primary prevention, including 7447 participants) [39] and in the CORDIOPREV trial (secondary prevention, including 1002 participants) [41]. In the PREDIMED randomized trial, 288 cases of the primary cardiovascular endpoint (myocardial infarction, stroke, or cardiovascular death) among 7447 randomly assigned participants were included in the analyses. In the CORDIOPREV randomized trial, 198 cases of the primary endpoint (including myocardial infarction, revascularization, ischemic stroke, peripheral artery disease, or cardiovascular death) among 1002 patients with previous coronary artery disease were included in the analyses. No adjustment was made to the Kaplan-Meier curves, given the randomized design. Results in Cox models did not materially change after a variety of adjustments. Reproduced with permission from references [39,41]. EVOO, extra-virgin olive oil; MedDiet: Mediterranean diet; PREDIMED, prevención con dieta Mediterránea; CORDIOPREV, Coronary Diet Intervention With Olive Oil and Cardiovascular Prevention.

The results reported by the PREDIMED trial for primary prevention of CVD were subsequently replicated in an independent trial for secondary prevention, known as CORDIOPREV (Coronary Diet Intervention With Olive Oil and Cardiovascular Prevention), a single-center RCT conducted in Córdoba, Spain. CORDIOPREV randomly assigned 1002 patients with established CAD (aged 20–75 y) to a MedDiet supplemented with EVOO or to a low-fat diet (control group), with a mean follow-up of 7 y. Compared to the control group, the intervention group experienced a 26% (95% CI: 8, 51%) relative reduction in the primary composite cardiovascular endpoint (including myocardial infarction, revascularization, ischemic stroke, peripheral artery disease, and cardiovascular death) [41]. (Figure 3 [39,41]). A smaller secondary prevention trial conducted in France, the Lyon Diet Heart Study (*n* = 605), found that a Mediterranean-type diet rich in α-linolenic acid (mainly from rapeseed oil) significantly reduced mortality and recurrences of myocardial infarction compared to the control group (usual diet) at 27 mo and 46 mo of follow-up [42].

PREDIMED-Plus is another large Spanish multi-center RCT with 23 recruitment centers evaluating the effects of a comprehensive lifestyle intervention including an energy-reduced MedDiet, promotion of physical activity, and behavioral support for weight loss (intervention group) as compared to a control group following only an ad libitum traditional MedDiet (without any caloric restriction). It randomly assigned 6874 participants, males aged 55–75 y and females aged 60–75 y, without CVD at baseline who had an initial BMI of 27–40 and metabolic syndrome [43]. The final results on the primary endpoint (a composite of nonfatal stroke, nonfatal myocardial infarction, or cardiovascular death) are anticipated in the near future. Favorable changes in the main cardiovascular disease risk factors after 1-y for the intervention group as compared to the control group were reported in an interim analysis [43]. Another interim analysis in a subsample of 1521 individuals found significant reductions in total fat mass and visceral fat mass and an increase in total lean mass in the intervention group compared to the control group after 3 y [44].

Low to moderate wine consumption is typically included in the definition of the MedDiet. Numerous large and well-conducted prospective cohort studies have demonstrated an inverse association between moderate alcohol intake and reduced CVD risk, particularly for wine consumed with meals [45,46]. A key component of the MedDiet score as a predictor of lower mortality in a Greek population was moderate consumption of alcohol [47]. Of note, in the traditional MedDiet, wine is often consumed with meals. However, the potential benefits of moderate alcohol consumption against CVD were not supported by several Mendelian randomization studies using genetic variants associated with alcohol intake or metabolism to minimize confounding and reverse causation in observational studies [48]. It should be noted that Mendelian randomization analyses cannot capture drinking patterns, and their underlying assumptions may not always hold. Nonetheless, there is an urgent need to obtain more robust evidence to address the current debate on alcohol abstention compared with moderation through an RCT. Recently, the European Research Council funded the University of Navarra Alumni Trialist Initiative (UNATI), a large pragmatic RCT to assess the health effects of alcohol. The design is a 4-y noninferiority trial randomizing >10,000 drinkers (males 50–70 y or females 55–75 y consuming ≥3 but ≤40 drinks/wk) living in Spain to receive intensive advice on each of these 2 options: abstention compared with moderate consumption of red wine (≤1 glass/d in females or ≤2 glasses/d in males) with meals. The primary endpoint is a composite of all-cause mortality, CVD, invasive cancer, liver cirrhosis, and other relevant outcomes [49]. This large RCT will generate more definitive data on the health effects of moderate alcohol consumption compared to abstention.

### Nutrition omics studies

Recent advances in omics technologies, such as genomics, metabolomics, proteomics, and metagenomics, have enabled the development of objective dietary biomarkers and the exploration of biological mechanisms underlying diet-disease relationships, facilitating precision nutrition research and practice. The MedDiet has been extensively studied for its impact on metabolomics and the gut microbiome, with significant implications for cardiometabolic health. Metabolomics, a tool for profiling small-molecule metabolites in biospecimen samples, provides a comprehensive overview of an individual’s metabolic status. These approaches have revealed not only distinct metabolomic signatures associated with adherence to the MedDiet, reflecting dietary intake and the body’s metabolic responses [50], but also effect modification by the gut microbiome [51]. In studies nested within the PREDIMED trial, and replicated in United States cohorts (NHS, NHS II, HPFS), a metabolite signature of 67 plasma metabolites was identified, significantly correlating with MedDiet adherence and predicting CVD risk in both Spanish and United States populations [52]. Extensive studies have been conducted to identify metabolomics markers of cardiometabolic diseases using blood samples from the PREDIMED trial, and key findings were summarized in a recent review article [50]. For example, plasma concentrations of branched-chain amino acids (leucine, isoleucine, and valine) were significantly associated with increased CVD risk, and these associations were mitigated by the MedDiet interventions [53]. Elevated short- and medium-chain acylcarnitines were also linked to higher CVD risk, and this association was attenuated with the MedDiet interventions. Plasma tryptophan was inversely associated with CVD risk, whereas kynurenic acid was positively associated with risk. Similarly, plasma glutamine predicted lower CVD risk, whereas glutamate was associated with a higher risk of CVD and heart failure. Reduced concentrations of arginine, a precursor for nitric oxide, were associated with increased CVD risk. In addition, a positive association between baseline plasma ceramide concentrations and incident CVD was observed, and the MedDiet intervention mitigated the deleterious effects of elevated plasma ceramide concentrations on CVD [54]. Cholesterol esters, polyunsaturated phosphatidylcholines, and certain triacylglycerols were inversely associated with CVD risk. Urinary tartaric acid concentrations, an objective measure of wine consumption, were associated with reduced CVD risk [55].

The MedDiet also influences the gut microbiome, with long-term adherence associated with specific taxonomic and functional microbial components. An analysis conducted in a subcohort of HPFS suggested that the protective associations of the MedDiet with cardiometabolic risk were both mediated through and modified by gut microbiome composition, with a stronger association observed among individuals with lower abundance of *Prevotella copri* [51]. In the PREDIMED-Plus trial [56], a 1-y energy-reduced MedDiet combined with physical activity significantly altered gut microbiota composition and fecal metabolites. The favorable influence of the MedDiet on the gut microbiome is attributed to its high content of fiber, polyphenols, and unsaturated fatty acids, as well as its lower intake of processed and red meats, although the effects of diet cannot completely be separated from increased physical activity [57]. Dietary fibers serve as substrates for bacterial fermentation, producing short-chain fatty acids such as butyrate, propionate, and acetate, which are likely to exert anti-inflammatory actions and contribute to gut barrier integrity. Polyphenols, abundant in fruits, vegetables, and EVOO, act as prebiotics, promoting the growth of beneficial bacteria whereas inhibiting pathogenic strains [58,59]. In addition, the high intake of long-chain ω-3 fatty acids in the MedDiet may reduce gut inflammation and modulate microbial diversity, further contributing to a healthier gut environment. However, functional understanding of these relationships remains limited due to variability in study designs and small sample sizes of most studies.

### Environmental sustainability of MedDiet

Accelerating changes in climate have brought attention to the MedDiet for its potential to mitigate these trends and other intertwined, adverse environmental effects of our current food systems [60]. Climate change, manifesting as extreme heat in some regions, droughts, floods, and fires, is particularly concerning because our Earth’s systems are reaching tipping points that become irreversible and produce vicious circles. Examples include the melting of Arctic permafrost and the Arctic icecap, which releases stored methane that adds further to global warming. These outcomes are not inevitable. Rapid action and global commitment are needed [61].

The largest contributor to climate change is the increase in greenhouse gas emissions (GHGe) due to the burning of fossil fuels, which must end as quickly as possible to limit severe consequences. The majority of these emissions are related to transportation, energy production, and industrial uses. However, the global food systems contribute ∼30% of GHGe from 3 major sources: direct emissions from food production; energy for fertilizer production, transportation, and refrigeration; and conversion of forests for cultivation and grazing to feed the growing global population’s diets that are increasingly animal-based [62]. Production of animal-sourced foods contributes most to these GHGe; for example, emissions are ∼160 times greater per serving of beef or sheep compared to a serving of legumes due to the inefficiency of converting feed to edible meat and the large amounts of methane produced by ruminant animals during digestion [63]. Production of dairy foods and pork results in fewer GHGe per serving, but this is still many times greater than that of nuts, soy, or other legumes.

The MedDiet has lower environmental impacts compared to diets of northern Europe and most other industrialized countries because of the limited amounts of animal-sourced foods. In a modeling analysis, Tilman and Clark [64] estimated that the adoption of the MedDiet pattern would be associated with much lower GHGe and land use conversion than current global dietary trends. As expected, adoption of a more strictly vegetarian diet would have even lower environmental impacts. Halting deforestation is particularly important because it avoids the release of methane, preserves a critical carbon sink, and supports water supplies and biodiversity. Any decrease in land use by growing foods for humans rather than for animals to produce milk and meat with huge inefficiencies can provide a double win because the released land can be used to capture carbon by reforestation. Even within Spain, compared to current diets, the traditional MedDiet pattern could potentially decrease GHGe by 72% and land use by 58% [65].

In addition to the plant-forward character of the MedDiet with its reduced reliance on animal-sourced foods, the hundreds of millions of olive trees in the greater Mediterranean that yield the principal source of fats and oils in the region’s traditional dietary pattern are central to the sustainability profile of the MedDiet. As a permanent crop, olive groves sequester carbon, preserve biodiversity, are drought tolerant, and are a barrier to desertification. In parallel, the prominence of tree nuts and tree fruit (also permanent crops) and legumes (which fix nitrogen and replenish soil health) in the MedDiet additionally contributes to its sustainability.

The EAT-Lancet Commission, charged with finding a pathway to feed the growing global population a diet that is both healthy and sustainable, conducted a detailed review of major food components to create the planetary health diet (PHD) [63]. This “bottoms up” approach resulted in a pattern that aligned almost perfectly with the traditional MedDiet and was applicable to food cultures around the world. Within the United States population, compared to the bottom quintile in adherence to the PHD pattern, being in the top quintile was associated with 30% lower GHGe from diet and 50% lower land use. Within the PREDIMED-Plus trial, using methods from the EAT-Lancet report, participants randomly assigned to a MedDiet pattern had reductions in GHGe, land use, energy consumption, and ocean acidification and eutrophication compared to the control group [66].

## Translation of MedDiet into Practice and Actions

The diversity and appeal of healthy food and cooking practices across the Mediterranean, as broadly represented by the MedDiet pyramid, is notable [67]. At the center of this is the Mediterranean plant-forward or plant-centric culinary tradition [[68], [69], [70], [71]], shaped over centuries by agricultural practices, regional biodiversity, and cultural exchange [72]. In Greece [69], a prime example is horta, or boiled sweet wild greens (often gathered in the mountains), served with EVOO. In southern Italy [73], orecchiette with broccoli rabe combines leafy greens and whole grain pasta, also served with EVOO, highlighting the nutritional synergy of fiber, polyphenols, and healthy fats. Following the Colombian exchange, tomatoes became a key element of Mediterranean cuisine, particularly in southern Italy [73], and Spain [74], e.g., in the tomato-based soups—gazpacho and salmorejo—of Andalusia in the south of Spain. Eggplant is one of the ingredients that distinguishes Turkish mezze [75], typically a series of small plates based on vegetable- and legume-sourced ingredients. Legumes or pulses are central to the whole of traditional Mediterranean cooking including the kitchens of the Eastern Mediterranean [[75], [76], [77], [78]] and the Middle East [79,80], as well as North Africa [81,82], as highlighted in such dishes as the vegetable tagines with chickpeas of Tunisia and Morocco, the red lentil soups of Egypt and Turkey, and the oven-roasted Gigante beans of Greece. Nuts, including almonds, hazelnuts, pine nuts, pistachios, and walnuts, are integral to the Mediterranean cuisine [68]. Plant-based ingredients also serve as the foundation of flavor development across the region, including pounded sauces, dips, and spreads, such as romesco, hummus, and pesto. Sofrito, the aromatic base of many dishes in Spain and elsewhere, typically combines onions, garlic, peppers, and other vegetables with EVOO.

The MedDiet culture bridges the past and the present by preserving traditional food practices while adapting to modern lifestyles. The health benefits associated with the MedDiet can be partly attributed to the incorporation of traditional foods, which are distinguished by their unique food compositions, often relying on locally sourced ingredients, and time-honored production and processing methods. This dietary pattern promotes a harmonious relationship between people and the natural environment [68,83].

However, this tradition is increasingly at risk due to changes in eating habits and the globalized food system, which favors convenience and highly processed foods over traditional practices [84]. In Mediterranean countries, particularly among younger generations, adherence to the traditional MedDiet has significantly declined, with a shift to a more Westernized dietary pattern characterized by increased consumption of sugar-sweetened beverages, fast food, and other ultra-processed foods and a reduction in consumption of vegetables, legumes, and whole grains [85,86]. This trend is driven by changes in food environments, urbanization, and the widespread availability and accessibility of inexpensive and highly marketed products. As a result, there has been a rising prevalence of obesity and metabolic disorders in Mediterranean populations, particularly among children and adolescents [87,88].

One example of this dietary shift is the region’s decline in carbohydrate quality over many decades (as has been the case in much of the world), a negative trend further exacerbated by increasing sedentary lifestyles [89]. This underscores the need for increasing consumption of whole, intact grains and slow-metabolizing, minimally processed carbohydrate-rich foods over highly processed refined starches. It is encouraging that the Mediterranean region is witnessing a modest resurgence of interest in whole grains, with some traditional practices being revived, adapted, or just actively preserved. For instance, in Crete, traditional dakos - whole grain barley rusks, rehydrated with water and topped with chopped tomatoes, feta cheese, and EVOO, have gained popularity [69]. Other traditional whole grains, such as farro and bulgur (cracked wheat) and pasta made with whole grains and legume flour (with pasta, more generally, best cooked *al dente* to slow digestion and absorption and improve glycemic response), are also gaining popularity.

Preserving the MedDiet traditions requires comprehensive efforts, including nutrition education to increase awareness of traditional foods, especially among the younger generations, strengthening local food systems to ensure access to fresh ingredients, and supporting sustainable food practices and policies. Since the early 1990s, many institutions and organizations have led educational efforts to increase public and professional literacy in the MedDiet and healthy Mediterranean culinary strategies. In the past 20 y, the Culinary Institute of America and the Department of Nutrition at Harvard T.H. Chan School of Public Health have collaborated on initiatives inspired by the MedDiet, including Healthy Kitchens, Healthy Lives [90], and Menus of Change National Leadership Summit [91]. These programs have engaged diverse audiences ranging from chefs, food business leaders, and K-12 school food service directors to physicians and other healthcare professionals, with the goal of promoting the integration of MedDiet principles into culinary education, healthcare, and the broader food system.

Building on these efforts, several international collaborations have emerged to further promote the MedDiet and its role in public health and sustainability. The Torribera Mediterranean Center*,* a collaborative initiative between the Culinary Institute of America and the University of Barcelona*,* organizes *Tomorrow Tastes Mediterranean* [92], an annual conference alternating between Spain and Greece that fosters global dialogs about healthy, sustainable food systems with special focus on the MedDiet. In addition, within the framework of a memorandum of understanding between the Academy of Athens’ Center for Public Health Research and Education and Department of Environmental Health at Harvard T.H. Chan School of Public Health, discussions on the MedDiet were organized at the Delphi Economic Forum [93]. Furthermore, the Mediterranean Agronomic Institute [94] has been actively engaged in promoting the MedDiet through research, education, and policy initiatives.

Now more than ever, as work continues to translate the framework of a PHD (including the MedDiet) into action and implementation, a critical need exists to emphasize the value of cross-disciplinary and cross-cultural collaboration, which is essential to elevate food choices, menus, dietary patterns and food systems that are both accessible and appealing while aligning with global health and sustainability priorities [95]. For example, if the flavor and quality of key ingredients of the MedDiet, such as fruits and vegetables, are diminished due to production and distribution demands of increasingly complex supply chains, the broader public health mission may be compromised. Recognizing and prioritizing palatability and enjoyment of foods and meals is not just a culinary concern but also an important public policy imperative that supports long-term dietary adherence and health outcomes.

Policies are essential to address socioeconomic barriers to promote healthy eating patterns and increase broader accessibility and affordability of healthy foods. The MedDiet has been widely incorporated into national and international dietary recommendations and clinical guidelines. In 2015, the *dietary guidelines advisory committee* in the United States recognized, for the first time, the MedDiet as one of the healthy eating patterns for reducing risk of chronic diseases [96]. Beyond its scientific and policy recognition, the MedDiet has also gained widespread popularity in the general public. In 2025, a panel of nutrition scientists and public health experts, convened by United States News & World Report, once again ranked the MedDiet as the #1 overall dietary pattern for the eighth consecutive year [97].

## Cultural Adaptation of the MedDiet

One of the strengths of the MedDiet is its versatility due to the diverse culinary traditions across the Mediterranean regions. Although the core principles of the diet remain consistent, variations in ingredients and cooking techniques reflect local agricultural practices, cultural influences, and historical trade routes [4]. One question that arises is the applicability of the MedDiet to other cultures beyond those of the Mediterranean regions. Although more limited research has been conducted on the health impacts of other traditional diets from, for example, Asia, Latin America, and Africa, the core principles of the MedDiet can readily be adapted to diverse food cultures whereas preserving their unique ingredients and flavors [16]. Future research may identify traditional dietary patterns that are optimally health-promoting in their own right, requiring no adaptation or influence from the MedDiet or any other dietary model. Dietary guidance should take into account cultural relevance by aligning with individuals’ food heritage, familiar flavors, and personal preferences to enhance acceptance, sustainability, and long-term adherence.

At the same time, although it is crucial to respect and preserve traditional diets, it is also important to avoid suggesting that individuals seeking healthier dietary choices should only consume foods from their own cultural heritage [95]. Nowadays, many people around the world appreciate and embrace a diverse range of cuisines from different cultures and geographical regions. This presents an opportunity to expand dietary choices inspired by diverse cultural models of healthy, sustainable eating patterns, as research continues to advance. A fusion or hybrid approach, such as, for example, healthy Asian-Mediterranean and Latin-African dietary patterns, can expand the range of options for individuals, improve diet quality, and promote personal and planetary health. This flexibility allows individuals to creatively tailor their diets to their personal and cultural preferences and health conditions, thus improving acceptance and longer-term adherence across diverse populations [98].

## Future Research Directions

Future directions in MedDiet research—and, indeed, research on all traditional dietary patterns—should emphasize multidisciplinary collaboration integrating nutrition epidemiology, basic and clinical research, anthropology, behavioral sciences, food and cultural studies, culinary science and practice, agriculture, sustainability, and policy. Large cohort studies and RCTs must extend beyond Mediterranean regions to assess effects across different genetic and cultural backgrounds. Behavioral and consumer insight strategies should focus on enhancing long-term adherence through technology, education, and community-based programs that can be scalable across large populations. Policies play a crucial role in addressing socioeconomic barriers to ensure the affordability of healthy foods while supporting the preservation of dietary traditions. Sustainability research should evaluate the environmental impact and practicality of adopting the MedDiet across different communities and countries, especially considering the expansion of olive cultivation in many countries well beyond the Mediterranean region. The adaptation of the MedDiet to non-Mediterranean countries, within the framework of planetary sustainability, has been termed the “Planeterranean” diet by the United Nations Educational, Scientific, and Cultural Organization [99,100]. To this end, nutrition interventions should incorporate diverse cultural models of healthy eating, including hybrid dietary patterns, to enhance adaptability and effectiveness.

The integration of multi-omic technologies is crucial for uncovering precise mechanisms underlying diet-disease relationships. In addition, mechanistic studies should explore the influence of the MedDiet on hallmarks of biological aging, such as slowing telomere shortening, mitigating epigenetic aging, improving mitochondrial function, maintaining protein homeostasis, and regulating nutrient-sensing pathways [101]. More research is needed to examine the effects of specific polyphenols from EVOO and other plant foods on gut microbiome composition, which may benefit brain health through the gut-brain axis by reducing neuroinflammation and oxidative stress. Additional research priorities include the development of more robust biomarkers for dietary intakes and predicting long-term cardiometabolic disease risk, the inclusion of diverse populations in clinical trials and nutrition omics studies, and the translation of research findings into clinical settings to improve personalized nutrition strategies. These efforts can help to deepen our understanding of the complex biological mechanisms underlying the health benefits of the MedDiet, solidifying its role in chronic disease prevention and management.

## Strengths and Limitations

This review provides a comprehensive overview of the history, evolution, and scientific advances of the MedDiet since the official publication of the MedDiet pyramid 30 y ago. It not only describes extensive epidemiologic, clinical trial, and mechanistic evidence but also discusses translation into practice, cultural adaptations, and environmental sustainability. Despite its broad scope and up-to-date nature, several limitations should be acknowledged. First, this is a narrative review rather than a systematic review, which would not be feasible given the wide scope of the topic. However, we have cited numerous systematic reviews and meta-analyses on the health effects of the MedDiet. Second, although much of the health-related evidence is observational, it is supported by several large RCTs. Third, when the MedDiet is examined in non-Mediterranean populations, the dietary pattern often differs, particularly due to limited olive oil consumption. Thus, “Mediterranean-Style diet” is a more appropriate term in these contexts. Finally, research on policy translation, cultural adaptation, and environmental sustainability of the MedDiet remains limited. Thus, further research is needed to evaluate these aspects.

In conclusion, since the official publication of the MedDiet pyramid in 1995, our understanding of the MedDiet has grown considerably, driven by advances in nutrition science, environmental health, and insights into its social-cultural impact. Over the past 3 decades, a substantial body of evidence has accumulated to strongly support the benefits of the MedDiet in preventing chronic diseases and promoting healthy aging. Recent findings have revealed its beneficial effects on the metabolome and the gut microbiome, providing novel insights into biological mechanisms underlying its health benefits. In practice, the MedDiet has expanded beyond the Mediterranean regions, adapting to different cultural and dietary contexts. The development of the MedDiet pyramid 30 y ago also inspired the creation of the Asian, African, and Latin American Heritage Diet Pyramids [16]. To maintain its relevance and impact, it is essential to address contemporary challenges such as climate change, unhealthy food environments, sedentary lifestyles, and rising obesity rates, as well as the diverse cultures and food systems of global populations.

## Author contributions

The authors’ responsibilities were as follows– FBH: drafted the initial manuscript; GD, AT, WCW, MAM-G: edited and contributed to its content; and all authors: read and approved the final manuscript.

## Funding

The “Prevención con Dieta Mediterránea” (PREDIMED) and PREDIMED-Plus trials were partially funded by the Spanish National Institutes of Health Carlos III. The PREDIMED-plus and University of Navarra Alumni Trialist Initiative (UNATI) studies received funding from the European Research Council (Advanced Research Grants). Additional financial support was provided by NIH grants R01HL118264, R01DK127601, and R01DK102896.

## Conflict of interest

The authors report no conflicts of interest.

## References

[1] Bach-Faig A., Berry E.M., Lairon D., Reguant J., Trichopoulou A., Dernini S. (2011). Mediterranean diet pyramid today. Science and cultural updates. Public Health Nutr.

[2] Trichopoulou A. (2021). Mediterranean diet as intangible heritage of humanity: 10 years on. Nutr. Metab. Cardiovasc. Dis..

[3] Dominguez L.J., Veronese N., Ragusa F.S., Petralia V., Ciriminna S., Di Bella G. (2024). Mediterranean diet and spirituality/religion: eating with meaning. Aging Clin. Exp. Res..

[4] Willett W.C., Sacks F., Trichopoulou A., Drescher G., Ferro-Luzzi A., Helsing E. (1995). Mediterranean diet pyramid: a cultural model for healthy eating. Am. J Clin. Nutr..

[5] Allbaugh L.G. (1953).

[6] Keys A. (1970). Coronary heart disease in seven countries. Circulation.

[7] Keys A. (1980).

[8] Report of the National Cholesterol Education Program expert panel on detection (1988). , evaluation, and treatment of high blood cholesterol in adults. The expert panel. Arch. Intern. Med..

[9] Pritikin N., McGrady P. (1982).

[10] Ornish D., Dr. Dean Ornish’s program for reversing heart disease (1995).

[11] Trichopoulou A., Kouris-Blazos A., Wahlqvist M.L., Gnardellis C., Lagiou P., Polychronopoulos E. (1995). Diet and overall survival in elderly people. BMJ.

[12] Trichopoulou A., Kouris-Blazos A., Vassilakou T., Gnardellis C., Polychronopoulos E., Venizelos M. (1995). Diet and survival of elderly Greeks: a link to the past. Am. J Clin. Nutr..

[13] Keys A. (1995). Mediterranean diet and public health: personal reflections. Am. J Clin. Nutr..

[14] Willett W.C. (1994). Diet and health: what should we eat?. Science.

[15] USDA (1992).

[16] LeBlanc K.E., Baer-Sinnott S., Lancaster K.J., Campos H., Lau K.H., Tucker K.L. (2024). Perspective: beyond the Mediterranean diet-exploring Latin American, Asian, and African heritage diets as cultural models of healthy eating. Adv. Nutr..

[17] GBD 2021 Adult BMI Collaborators (2025). Global, regional, and national prevalence of adult overweight and obesity, 1990-2021, with forecasts to 2050: a forecasting study for the Global Burden of Disease Study 2021. Lancet.

[18] GBD 2021 Adolescent BMI Collaborators (2025). Global, regional, and national prevalence of child and adolescent overweight and obesity, 1990-2021, with forecasts to 2050: a forecasting study for the Global Burden of Disease Study 2021. Lancet.

[19] Tobias D.K., Papatheodorou S., Yamamoto J.M., Hu F.B. (2023). A primer on systematic review and meta-analysis in diabetes research. Diabetes Care.

[20] Renaud S., de Lorgeril M., Delaye J., Guidollet J., Jacquard F., Mamelle N. (1995). Cretan Mediterranean diet for prevention of coronary heart disease. Am. J. Clin. Nutr..

[21] Trichopoulou A., Costacou T., Bamia C., Trichopoulos D. (2003). Adherence to a Mediterranean diet and survival in a Greek population, N Engl. J Med..

[22] Hu F.B., Willett W.C. (2001). Diet and coronary heart disease: findings from the Nurses’ Health Study and Health Professionals’ Follow-up Study, J Nutr. Health Aging..

[23] Hu F.B., Satija A., Rimm E.B., Spiegelman D., Sampson L., Rosner B. (2016). Diet assessment methods in the nurses’ health studies and contribution to evidence-based nutritional policies and guidelines, Am. J Public Health..

[24] Fung T.T., Rexrode K.M., Mantzoros C.S., Manson J.E., Willett W.C., Hu F.B. (2009). Mediterranean diet and incidence of and mortality from coronary heart disease and stroke in women. Circulation.

[25] Shan Z., Li Y., Baden M.Y., Bhupathiraju S.N., Wang D.D., Sun Q. (2020). Association between healthy eating patterns and risk of cardiovascular disease. JAMA Intern. Med..

[26] Shan Z., Wang F., Li Y., Baden M.Y., Bhupathiraju S.N., Wang D.D. (2023). Healthy eating patterns and risk of total and cause-specific mortality. JAMA Intern. Med..

[27] Sotos-Prieto M., Bhupathiraju S.N., Mattei J., Fung T.T., Li Y., Pan A. (2015). Changes in diet quality scores and risk of cardiovascular disease among US men and women. Circulation.

[28] Sotos-Prieto M., Bhupathiraju S.N., Mattei J., Fung T.T., Li Y., Pan A. (2017). Association of changes in diet quality with total and cause-specific mortality. N Engl. J. Med..

[29] Ahmad S., Demler O.V., Sun Q., Moorthy M.V., Li C., Lee I.M. (2020). Association of the Mediterranean diet with onset of diabetes in the women’s health study. JAMA Netw. Open.

[30] Zeraattalab-Motlagh S., Jayedi A., Shab-Bidar S. (2022). Mediterranean dietary pattern and the risk of type 2 diabetes: a systematic review and dose-response meta-analysis of prospective cohort studies. Eur. J. Nutr..

[31] Morze J., Danielewicz A., Przybyłowicz K., Zeng H., Hoffmann G., Schwingshackl L. (2021). An updated systematic review and meta-analysis on adherence to Mediterranean diet and risk of cancer. Eur. J. Nutr..

[32] Fu J., Tan L.J., Lee J.E., Shin S. (2022). Association between the Mediterranean diet and cognitive health among healthy adults: A systematic review and meta-analysis. Front. Nutr..

[33] Martínez-González M.A., Sayón-Orea C., Bullón-Vela V., Bes-Rastrollo M., Rodríguez-Artalejo F., Yusta-Boyo M.J. (2022). Effect of olive oil consumption on cardiovascular disease, cancer, type 2 diabetes, and all-cause mortality: A systematic review and meta-analysis. Clin. Nutr..

[34] Guasch-Ferré M., Liu G., Li Y., Sampson L., Manson J.E., Salas-Salvadó J. (2020). Olive oil consumption and cardiovascular risk in U.S. adults. J Am. Coll. Cardiol..

[35] Guasch-Ferré M., Hruby A., Salas-Salvadó J., Martínez-González M.A., Sun Q., Willett W.C. (2015). Olive oil consumption and risk of type 2 diabetes in US women. Am. J. Clin. Nutr..

[36] Guasch-Ferré M., Li Y., Willett W.C., Sun Q., Sampson L., Salas-Salvadó J. (2022). Consumption of olive oil and risk of total and cause-specific mortality among U.S. adults. J. Am. Coll. Cardiol..

[37] Tessier A.J., Cortese M., Yuan C., Bjornevik K., Ascherio A., Wang D.D. (2024). Consumption of olive oil and diet quality and risk of dementia-related death. JAMA Netw. Open.

[38] Zhang Y., Chadaideh K.S., Li Y., Li Y., Gu X., Liu Y. (2025). Butter and plant-based oils intake and mortality. JAMA Intern. Med..

[39] Estruch R., Ros E., Salas-Salvadó J., Covas M.I., Corella D., Arós F. (2018). Primary prevention of cardiovascular disease with a Mediterranean diet supplemented with extra-virgin olive oil or nuts. N Engl. J. Med..

[40] Martínez-González M.A., Salas-Salvadó J., Estruch R., Corella D., Fitó M., Ros E. (2015). Benefits of the Mediterranean diet: insights from the PREDIMED study. Prog. Cardiovasc. Dis..

[41] Delgado-Lista J., Alcala-Diaz J.F., Torres-Peña J.D., Quintana-Navarro G.M., Fuentes F., Garcia-Rios A. (2022). Long-term secondary prevention of cardiovascular disease with a Mediterranean diet and a low-fat diet (CORDIOPREV): a randomised controlled trial. Lancet.

[42] de Lorgeril M., Salen P., Martin J.L., Monjaud I., Delaye J., Mamelle N. (1999). Mediterranean diet, traditional risk factors, and the rate of cardiovascular complications after myocardial infarction: final report of the Lyon Diet Heart Study. Circulation.

[43] Sayón-Orea C., Razquin C., Bulló M., Corella D., Fitó M., Romaguera D. (2019). Effect of a nutritional and behavioral intervention on energy-reduced Mediterranean diet adherence among patients with metabolic syndrome: interim analysis of the PREDIMED-plus randomized clinical trial. JAMA.

[44] Konieczna J., Ruiz-Canela M., Galmes-Panades A.M., Abete I., Babio N., Fiol M. (2023). An energy-reduced Mediterranean diet, physical activity, and body composition: an interim subgroup analysis of the PREDIMED-plus randomized clinical trial. JAMA Netw. Open.

[45] Gea A., Bes-Rastrollo M., Toledo E., Garcia-Lopez M., Beunza J.J., Estruch R. (2014). Mediterranean alcohol-drinking pattern and mortality in the SUN (Seguimiento Universidad de Navarra) Project: a prospective cohort study. Br. J Nutr.

[46] Jani B.D., McQueenie R., Nicholl B.I., Field R., Hanlon P., Gallacher K.I. (2021). Association between patterns of alcohol consumption (beverage type, frequency and consumption with food) and risk of adverse health outcomes: a prospective cohort study. BMC Med.

[47] Trichopoulou A., Bamia C., Trichopoulos D. (2009). Anatomy of health effects of Mediterranean diet: Greek EPIC prospective cohort study. BMJ.

[48] Kassaw N.A., Zhou A., Mulugeta A., Lee S.H., Burgess S., Hyppönen E. (2024). Alcohol consumption and the risk of all-cause and cause-specific mortality-a linear and nonlinear Mendelian randomization study. Int. J. Epidemiol..

[49] Martínez-González M.A. (2024). Should we remove wine from the Mediterranean diet?: a narrative review. Am. J. Clin. Nutr..

[50] Martínez-González M.A., Planes F.J., Ruiz-Canela M., Toledo E., Estruch R., Salas-Salvadó J. (2025). Recent advances in precision nutrition and cardiometabolic diseases. Rev. Esp. Cardiol. (Engl. Ed)..

[51] Wang D.D., Nguyen L.H., Li Y., Yan Y., Ma W., Rinott E. (2021). The gut microbiome modulates the protective association between a Mediterranean diet and cardiometabolic disease risk. Nat. Med..

[52] Li J., Guasch-Ferré M., Chung W., Ruiz-Canela M., Toledo E., Corella D. (2020). The Mediterranean diet, plasma metabolome, and cardiovascular disease risk. Eur. Heart J..

[53] Ruiz-Canela M., Toledo E., Clish C.B., Hruby A., Liang L., Salas-Salvadó J. (2016). Plasma branched-chain amino acids and incident cardiovascular disease in the PREDIMED trial. Clin. Chem..

[54] Wang D.D., Toledo E., Hruby A., Rosner B.A., Willett W.C., Sun Q. (2017). Plasma ceramides, Mediterranean diet, and incident cardiovascular disease in the PREDIMED trial (prevención con dieta mediterránea). Circulation.

[55] Domínguez-López I., Lamuela-Raventós R.M., Razquin C., Arancibia-Riveros C., Galkina P., Salas-Salvadó J. (2025). Urinary tartaric acid as a biomarker of wine consumption and cardiovascular risk: the PREDIMED trial. Eur. Heart J..

[56] Martínez-González M.A., Buil-Cosiales P., Corella D., Bulló M., Fitó M., Vioque J. (2019). Cohort Profile: design and methods of the PREDIMED-Plus randomized trial. Int. J Epidemiol..

[57] García-Gavilán J.F., Atzeni A., Babio N., Liang L., Belzer C., Vioque J. (2024). Effect of 1-year lifestyle intervention with energy-reduced Mediterranean diet and physical activity promotion on the gut metabolome and microbiota: a randomized clinical trial. Am. J. Clin. Nutr..

[58] Gundogdu A., Nalbantoglu O.U. (2023). The role of the Mediterranean diet in modulating the gut microbiome: a review of current evidence. Nutrition.

[59] Merra G., Noce A., Marrone G., Cintoni M., Tarsitano M.G., Capacci A. (2020). Influence of Mediterranean diet on human gut microbiota. Nutrients.

[60] Dernini S., Berry E.M. (2015). Mediterranean diet: from a healthy diet to a sustainable dietary pattern. Front Nutr.

[61] Atwoli L., Baqui A.H., Benfield T., Bosurgi R., Godlee F., Hancocks S. (2021). Call for emergency action to limit global temperature increases, restore biodiversity, and protect health, N Engl. J Med..

[62] Xu X., Sharma P., Shu S., Lin T.S., Ciais P., Tubiello F.N. (2021). Global greenhouse gas emissions from animal-based foods are twice those of plant-based foods. Nat. Food..

[63] Willett W., Rockström J., Loken B., Springmann M., Lang T., Vermeulen S. (2019). Food in the Anthropocene: the EAT-Lancet Commission on healthy diets from sustainable food systems. Lancet.

[64] Tilman D., Clark M. (2014). Global diets link environmental sustainability and human health. Nature.

[65] Sáez-Almendros S., Obrador B., Bach-Faig A., Serra-Majem L. (2013). Environmental footprints of Mediterranean versus Western dietary patterns: beyond the health benefits of the Mediterranean diet. Environ. Health..

[66] Álvarez-Álvarez L., Vitelli-Storelli F., Rubín-García M., García S., Bouzas C., Ruíz-Canela M. (2024). Impact of Mediterranean diet promotion on environmental sustainability: a longitudinal analysis. Public Health.

[67] Trichopoulou A. (2012). Diversity v. globalization: traditional foods at the epicentre. Public Health Nutr.

[68] Jenkins N.H. (2008).

[69] Wolfert P. (1998).

[70] Goldstein J. (2008).

[71] Kremezi A., De Los Santos P. (2014).

[72] Braudel F. (2023).

[73] Jenkins N.H. (2007).

[74] Roden C. (2012).

[75] Dagdeviren M. (2019).

[76] Kremezi A. (2018).

[77] Kochilas D. (2012).

[78] Zaytinya A.J. (2024).

[79] Roden C. (2000).

[80] Ottolenghi Y., Tamimi S. (2012).

[81] Latta H.B.R. (2023).

[82] Wolfert P. (2011).

[83] Trichopoulou A., Soukara S., Vasilopoulou E. (2007). Traditional foods: a science and society perspective. Trends Food Sci. Technol..

[84] Ambikapathi R., Schneider K.R., Davis B., Herrero M., Winters P., Fanzo J.C. (2022). Global food systems transitions have enabled affordable diets but had less favourable outcomes for nutrition, environmental health, inclusion and equity. Nat. Food..

[85] Rosi A., Scazzina F., Giampieri F., Abdelkarim O., Aly M., Pons J. (2024). Adherence to the Mediterranean diet in 5 Mediterranean countries: A descriptive analysis of the DELICIOUS project, Mediterr. J Nutr. Metab..

[86] Šarac J., Havaš Auguštin D., Lovrić M., Stryeck S., Šunić I., Novokmet N. (2021). A generation shift in Mediterranean diet adherence and its association with biological markers and health in Dalmatia, Croatia. Nutrients.

[87] Grosso G., Galvano F. (2016). Mediterranean diet adherence in children and adolescents in southern European countries. NFS J.

[88] López-Gil J.F., García-Hermoso A., Martínez-González M.Á., Rodríguez-Artalejo F. (2024). Mediterranean diet and cardiometabolic biomarkers in children and adolescents: A systematic review and meta-analysis. JAMA Netw. Open.

[89] Ludwig D.S., Hu F.B., Tappy L., Brand-Miller J. (2018). Dietary carbohydrates: role of quality and quantity in chronic disease. BMJ.

[90] Healthy Kitchens, Healthy Lives: Healthy Kitchens, Healthy Lives [Internet]; [cited March 19, 2025]. Available from: https://www.healthykitchens.org/.

[91] Menus of Change: Menus of Change [Internet]; [cited March 19, 2025]. Available from: https://www.menusofchange.org/.

[92] Tomorrow Tastes Mediterranean International Conference (2025). Kalamata and the region of Messinia, Greece [Internet]. https://tomorrowtastesmediterranean.org/.

[93] Delphi Economic Forum: Delphi Economic Forum X [Internet]; [cited March 19, 2025]. Available from: https://delphiforum.gr/.

[94] CIHEAM: International Center for Advanced Mediterranean Agronomic Studies [Internet]; [cited March 19, 2025]. Available from: https://www.ciheam.org/.

[95] Biesbroek S., Kok F.J., Tufford A.R., Bloem M.W., Darmon N., Drewnowski A. (2023). Toward healthy and sustainable diets for the 21st century: importance of sociocultural and economic considerations. Proc. Natl. Acad. Sci. U. S. A..

[96] (2015). USDA. Scientific report of the 2015 Dietary Guidelines Advisory Committee.

[97] Find the best diet for you [Internet]; [cited March 19, 2025]. Available from: https://health.usnews.com/best-diet.

[98] Hu F.B. (2024). Diet strategies for promoting healthy aging and longevity: an epidemiological perspective. J Intern. Med..

[99] Vetrani C., Piscitelli P., Muscogiuri G., Barrea L., Laudisio D., Graziadio C. (2022). ‘Planeterranea’: an attempt to broaden the beneficial effects of the Mediterranean diet worldwide. Front Nutr.

[100] Colao A., Vetrani C., Muscogiuri G., Barrea L., Tricopoulou A., Soldati L. (2022). “Planeterranean” Diet: extending worldwide the health benefits of Mediterranean Diet based on nutritional properties of locally available foods. J. Transl. Med..

[101] Shannon O.M., Ashor A.W., Scialo F., Saretzki G., Martin-Ruiz C., Lara J. (2021). Mediterranean diet and the hallmarks of ageing. Eur. J. Clin. Nutr..

